# Small-Volume Flow Cytometry-Based Multiplex Analysis of the Activity of Small GTPases

**DOI:** 10.1007/978-1-4939-8612-5_13

**Published:** 2018

**Authors:** Peter Simons, Virginie Bondu, Angela Wandinger-Ness, Tione Buranda

**Keywords:** Rho GTPase, Rab GTPase, Cell signaling, Cytoskeleton, Hantavirus, Flow cytometry, Integrin activation, Sepsis, Multiplex, Protease-activated receptors (PARs), Thrombin, Argatroban, Bead functionalization, Glutathione-*S*-transferase (GST), GTPase effector beads, Rap1, RhoA, Rac1, Rab7, Fluorescence calibration beads

## Abstract

Small, monomeric guanine triphosphate hydrolases (GTPases) are ubiquitous cellular integrators of signaling. A signal activates the GTPase, which then binds to an effector molecule to relay a signal inside the cell. The GTPase effector trap flow cytometry assay (G-Trap) utilizes bead-based protein immobilization and dual-color flow cytometry to rapidly and quantitatively measure GTPase activity status in cell or tissue lysates. Beginning with commercial cytoplex bead sets that are color-coded with graded fluorescence intensities of a red (700 nm) wavelength, the bead sets are derivatized to display glutathione on the surface through a detailed protocol described here. A different glutathione-*S*-transferase-effector protein (GST-effector protein) can then be attached to the surface of each set. For the assay, users can incubate bead sets individually or in a multiplex format with lysates for rapid, selective capture of active, GTP-bound GTPases from a single sample. After that, flow cytometry is used to identify the bead-borne GTPase based on red bead intensity, and the amount of active GTPase per bead is detected using monoclonal antibodies conjugated to a green fluorophore or via labeled secondary antibodies. Three examples are provided to illustrate the efficacy of the effector-functionalized beads for measuring the activation of at least five GTPases in a single lysate from fewer than 50,000 cells.

## 1 Introduction

Members of the Ras-related superfamily of small, monomeric GTPases, including Rho, Ras, and Rab subfamilies, serve as critical integrators of cellular functions from cell division and survival to membrane trafficking [1–6]. Genetic diseases and infectious agents such as viruses and bacteria are known to co-opt the signaling functions of GTPases, making GTPases attractive diagnostic and therapeutic targets [7–15]. Current methods for measuring the activation status of small GTPases rely on glutathione bead-based effectorpull-down/immunoblot assays, and ELISA-based effector-binding assay kits. The significant shortcomings of these methods are that they are labor intensive and require large sample sizes, purified effector proteins, or expensive kits. Additionally, sample processing times are critical because of the lability of the GTP-bound state due to hydrolysis. Here we describe the GTPase activity assay platform (G-Trap) [16], a multiplex, bead-based effector-binding assay that can rapidly monitor the activation status of multiple GTPases from a single-cell lysate [16, 17].

The GST-effector proteins consisting of the minimal GTPase-binding domains (RBD) for the studies are PAK-1 RBD (a Rac1 and Cdc42 effector), Raf-1 RBD (a Ras effector), Rhotekin-RBD (a Rho effector), RalGDS-RBD (a RAP1 effector protein), and RILP-RBD (a Rab7 effector) [16, 17]. Beads for each target effector (10,000/target) are mixed and added to cell lysates typically generated from 50,000 cells. The beads are incubated with cell lysates for 1 h at 4 °C, centrifuged, and resuspended in 50 μL of buffer (1:20 final antibody dilution). Monoclonal antibodies for each target GTPase are pooled and added to the multiplex bead suspension and incubated for 1 h at 4 °C. A secondary antibody tagged with Alexa 488 dye is then used to label bead-associated antibodies fluorescently. The samples are then analyzed on a flow cytometer where the red fluorescence identifies the specific effector bead and is used to gate the green fluorescence and quantify the amount of each target, GTP-bound GTPase. We demonstrate the functionality of the assay in three tests involving the activation of multiple GTPases. The first example measures the signaling cascade of GTPases that are activated to allow β_3_ integrin-mediated cellular entry of Sin Nombre virus (SNV) in the course of a productive infection [16, 17]. The second example measures GTPase activity downstream of signaling of protease-activated receptors (PARs), after exposure to thrombin found in the plasma samples drawn from patients with hantavirus cardiopulmonary syndrome (HCPS) [18]. The third example measures GTPase activation due to bacterial factors [12, 19] present in a plasma sample from a septic patient. Control reagents used for these illustrative examples are given in Table 1.

**Fig. 1:**
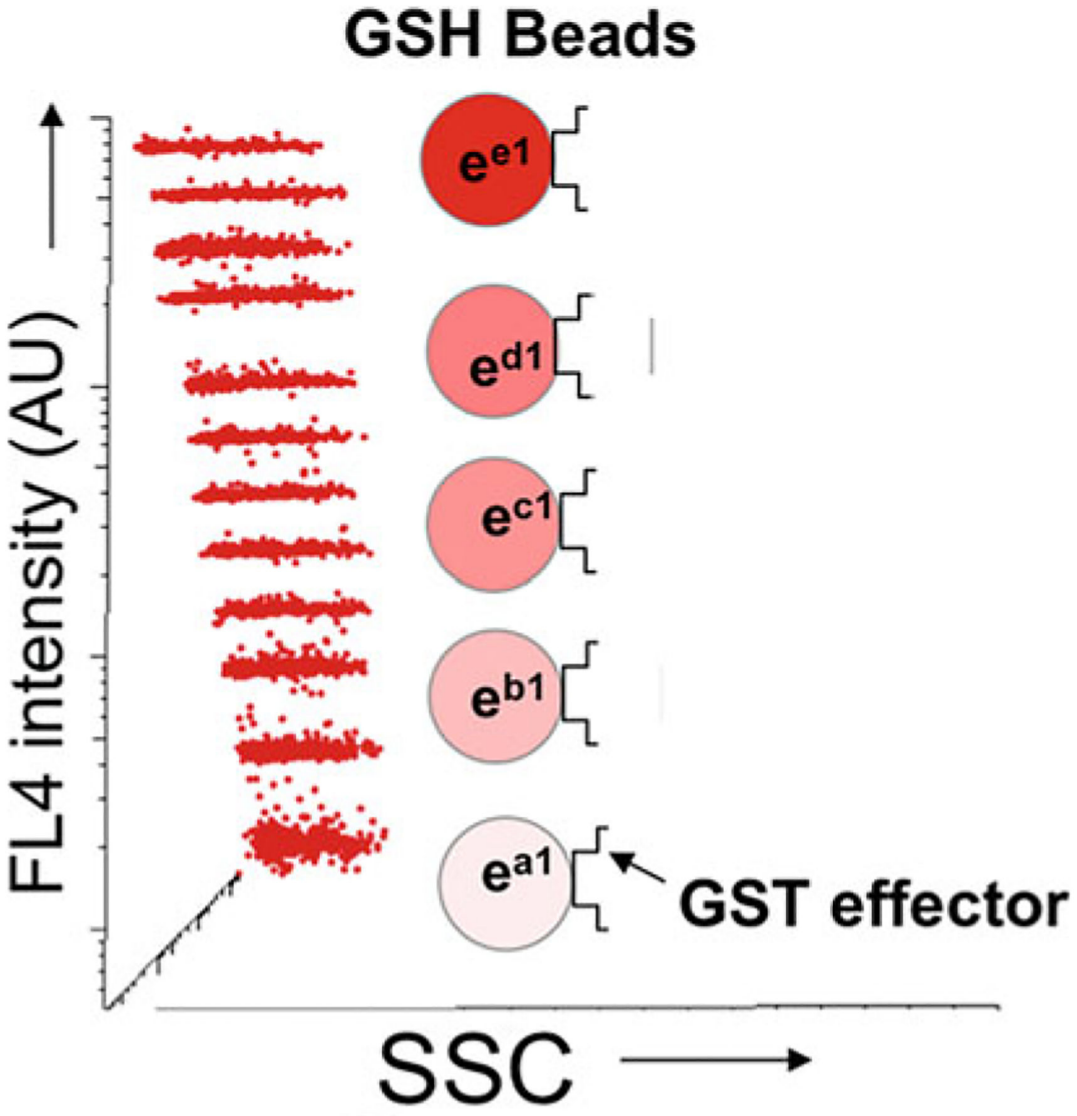
The GTPase effector trap flow cytometry assay (G-Trap). The plot of red fluorescence (FL4) versus side scatter (SSC) of a set of 12 of Cyto-Plex™ beads dyed with 12 discrete levels of 700 nm fluorescence. In the G-Trap assay, the letters *a*, *b*, *c*, *d*, and *e* identify and link effectors, their cognate GTPases, and fluorescently labeled antibodies FL1 (520 nm emission) or FL2 (580 nm emission) used for readout. In single or multiplex format, glutathione bead populations coated with effectors are used to capture specific active GTPases. In multiplex format, the effector and GTPase identities are defined by the intensity level of red fluorescence encoded on each bead

It is well established that flow cytometry is an ideal platform for measuring multiple analytes, simultaneously using cytoplex bead populations encoded with fluorescent dyes of graded intensities, with which the bead is uniquely identified (*see* Fig. 1) [17, 20]. Flow cytometry is capable of exciting at multiple absorption bands and detecting fluorescence at different emission wavelengths, and it is then possible to detect various analytes, simultaneously from a single sample [21–25]. The commercially available cytoplex beads used by us have up to 12 different intensity levels that can be used as unique bead identifiers. Furthermore, the beads are currently available in two sizes that can be resolved by flow cytometry forward light scatter. Thus, up to 24 different analytes can be measured. Our experience to date suggests that a maximum of six targets at a time is optimal for reproducible assay results [17].

## 2 Materials

### 2.1 Microspheres, Supplies, and Equipment

1. Cyto-Plex™ far-red fluorescent carboxylated microspheres (beads), uniform 4–5 μm in diameter, 12 sets with 12 discrete dye levels, at 10^8^ beads/mL, 1 mL of each set (Thermo Fisher Scientific): We use the 5.4 μm sized beads.
2. Carboxyl polystyrene beads, 5% w/v, 10 mL (Spherotech): Our batch was 5.28 μm.
3. Amino polystyrene beads, 5% w/v, 10 mL (Spherotech): Our batch was 3.57 μm.
4. Quantum™ FITC MESF (Molecules of Equivalent Soluble Fluorochrome) beads, five sets of commercial beads in which each subset is functionalized with discrete titers of fluorescein conjugates (Bangs Labs).
5. Refrigerated microcentrifuge with swinging bucket rotor and 0.65 mL microcentrifuge tubes.
6. Flow cytometer with a far-red laser, such as an Accuri C6.
7. pH meter.
8. Rotator, nutator.
9. Nitrogen-bubbling apparatus.

### 2.2 Synthesis of Glutathione Beads

1. 1% (v/v) Tween-20 stock.
2. pH 6 buffer: 0.1 M 2-(4-morpholino)-ethane sulfonic acid (MES), pH 6.0, 0.15 M NaCl, 0.01% (v/v) Tween-20.
3. 1-Ethyl-3-(dimethylaminopropyl) carbodiimide hydrochloride (EDAC).
4. Sulfo-N-hydroxysuccinimide (sNHS).
5. Rinse solution: 0.15 M NaCl, 0.01% (v/v) Tween-20, with no pH buffer.
6. pH 8.4 buffer: 0.1 M NaHCO_3_, pH 8.4, 0.01% (v/v) Tween-20.
7. 2 M 1,6-diaminohexane (hexamethylenediamine), pH 8.4.
8. pH 7 buffer: 0.1 M Sodium phosphate, pH 7.0, 0.01% (v/v) Tween-20.
9. Bifunctional crosslinker: 0.2 M Sulfosuccinimidyl 4-[N-maleimidomethyl] cyclohexane-1-carboxylate (sSMCC) in dimethyl sulfoxide (DMSO). Store at −80 °C.
10. 0.2 M Reduced glutathione, pH 7.0: Store in 50 μL aliquots at −20 °C.
11. 5 mg/mL Alexa Fluor 488 NHS ester (Thermo Fisher Scientific) in DMSO: Store at −80 °C
12. A fusion protein such as glutathione-*S*-transferase-green fluorescent protein (GST-GFP) to measure glutathione on the GST-functionalized beads, or your laboratory’s GST fusion protein and a fluorescent detection agent that binds to it.

### 2.3 Cell Culture

The G-Trap assay is used to measure GTP loading of multiple GTPase targets, found in cell lysates [16, 17]. The reader may cultivate cells using applicable standard procedures for their target cells, and treat cells with known activators and inhibitors (*see* Table 1) [16, 17] to establish the applicability of the assay in the setting defined by the reader.

### 2.4 GST-Effector Protein Production

Following GST-effector chimeras are used for the studies described here:

1. p21 activated kinase protein-binding domain (PAK-1 PBD), a Rac1 effector (MilliporeSigma).
2. Raf-1 (v-raf-1 murine leukemia viral oncogene homolog 1) RBD, a Ras effector protein (MilliporeSigma).
3. Rhotekin-RBD, a Rho effector protein (Cytoskeleton).
4. Ral-GDS RBD, a Rap-1 effector protein (Thermo Fisher Scientific).
5. GST-RILP, prepared as previously described [16].

### 2.5 GTPase Assay

1. Antibodies: Monoclonal rabbit anti-Rap1 (Santa Cruz Bio-technology); monoclonal mouse antibodies anti-Rho (A, B, C) clone 55, anti-Rac1, anti-Rab7, and the secondary antibody goat anti-mouse IgG (H + L) conjugated to Alexa Fluor 488 (MilliporeSigma); monoclonal mouse anti-Ras antibody (Abcam).
2. Activators and inhibitors: Rap1 activator 8-Cpt-2me-cAMP (50 μM) (R&D Systems); Rac1 inhibitor NSC23766 (100 μM) and Rho activator calpeptin (1 μM) (Millipore-Sigma); recombinant epidermal growth factor (EGF) (10 nM) (Thermo Fisher Scientific); mP6, a myristoylated hexapeptide (myr-FEERA-OH), custom synthesized at New England Peptide, H-Ras and K-Ras inhibitor, FTI 277 (100 nM) (Tocris); Rap1 inhibitor (GGTI 298) (10 μM).
3. 2× RIPA buffer: 100 mM Tris–HCl, pH 7.4, 300 mM NaCl, 2 mM ethylenediaminetetraacetic acid (EDTA), 2 mM NaF, 2 mM Na_3_VO_4_, 2% (v/v) NP-40, 0.5% (w/v) sodium deoxycholate. Just before adding to the culture medium supplement with 2 mM phenylmethylsulfonyl fluoride (PMSF) and 2× protease inhibitors (Halt™ Protease and Phosphatase Cocktail # 78442, ThermoScientific).
4. HHB buffer: 7.98 g/L HEPES (Na salt), 6.43 g/L NaCl, 0.75 g/L KCl, 0.095 g/L MgCl_2_, and 1.802 g/L glucose.
5. HPSMT buffer (an intracellular mimic): 30 mM HEPES, pH 7.4, 140 mM KCl, 12 mM NaCl, 0.8 mM MgCl_2_, 0.01% (v/v) Tween-20.
6. Blocking buffer 1: 0.1% Bovine serum albumin (BSA) in HPSMT.
7. Blocking buffer 2: 5% BSA in HPSMT.
8. 10 mM EDTA and 0.2% sodium azide for use at 1:10 dilution in storage buffer.

## 3 Methods

### 3.1 Synthesis of Glutathione Beads

High-site-density glutathione-derivatized beads used for flow cytometry have been synthesized previously from 13 μm dextran-cross-linked agarose beads [26, 27], and 4 μm amino polystyrene beads [20]. In this method, 5.4 μm Cyto-Plex™ carboxylated polystyrene bead sets are first converted to amino beads, and then to glutathione beads. We use standard practices with high concentrations of reagents to obtain a high glutathione site density on the beads. A high surface coverage of glutathione (GSH) enables robust capture of soluble GST fusion proteins [28]. The 12 Cyto-Plex™ carboxyl bead sets are coded with 12 graded intensities of far-red fluorescence when excited at a fixed wavelength, which does not interfere with fluorescein or phycoerythrin fluorescence detection. We suggest the use of a centrifuge with a swinging-bucket rotor and slow deceleration for ease of removing 90% of the supernatant, without disturbing the beads, during the many centrifugations in this synthesis. All reactions are at room temperature. While the protocol below is written for Cyto-Plex™ beads, we used an inexpensive (nonfluorescent) set of polystyrene carboxyl beads for pilot-testing our protocol for optimizing the synthetic conversion of carboxyl- to amino-functional groups on the beads, and subsequent coupling to glutathione (*see* Note 1).

**Fig. 2:**
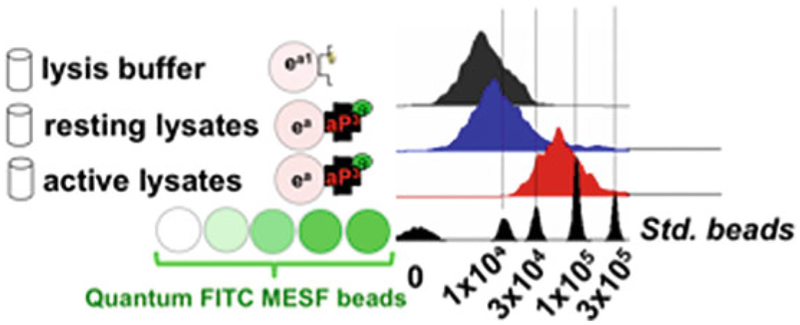
Single-analyte assay for RILP: GTP·Rab7 captured on beads. Fluorescently labeled detection antibody added to lysis buffer is used to assess nonspecific binding of the antibody to beads. Flow cytometry histograms of RILP-RBD effector beads incubated at 4 °C with resting HeLa cell lysates or with EGF-stimulated HeLa cell lysates show increased Rab7-GTP bound, the levels of which can be quantified using commercial standard calibration beads (Quantum™ FITC MESF). Quantum™ FITC MESF beads comprise five sets of distinct bead populations. Each bead population is distinguished by a discrete number of doped fluorophores of known calibration. The average fluorophores/bead on each bead population is shown on the *x*-axis. The calibration beads are used to quantify the occupancy of Rab7-specific antibodies on RILP-effector beads. After correcting for nonspecific binding, 7.1 ± 1.2 × 10^3^ Rab7-GTP molecules/bead were recovered in resting cell lysates, and 6.7 ± 0.3 × 10^4^ Rab7-GTP molecules/bead were retrieved in EGF-stimulated cell lysates [16]

1. A bead set in its bottle is rocked gently on its side for 2 min, rotated ¼ turn and rocked again for 2 min, and continued until the beads are in a milky suspension. 15 s of immersion in a low-power ultrasonic bath can help the resuspension.
2. Place 4 μL of 1% Tween-20 in a 0.65 mL centrifuge tube, add 400 μL of the bead suspension, mix gently with a pipette, and then allow the suspension to settle overnight to coat the beads and the tube with Tween-20, decreasing bead aggregation and adhesion to the tube (*see* Note 2).
3. Remove all but ~10 μL of the supernatant, resuspend the beads, and give two standard washes. For a regular wash, add 100 μL of pH 6 buffer to 10 μL of suspension, mix with a vortex mixer, centrifuge at 3–5000 × *g* for 2 min, remove 100 μL of supernatant, and resuspend the remaining 10 μL of beads with a vortex mixer. This standard wash assures that a nominal factor of 10× dilution of the undesired solute is achieved. Resuspension in minimal buffer ensures equal exposure of all beads to the next reagent.
4. Weigh 4 mg of EDAC and 8 mg of sNHS into a microfuge tube, add 100 μL of pH 6 buffer, immediately dissolve by vortexing, add this to a bead set, and mix. Place the microfuge tube in a rotator with a horizontal axis of rotation for 30 min to keep the beads in suspension, away from the tube lid and sides, while the site density of sNHS ester intermediate builds on the beads.
5. Centrifuge at 3–5000 × *g* for 2 min, remove all but 10 μL of the supernatant, resuspend the beads, and then wash two times with 100 μL of rinse solution, which will dilute the EDAC and sNHS while keeping the pH low and the sNHS ester intact.
6. Resuspend the beads in 180 μL of pH 8.4 buffer, immediately add 20 μL of 2 M 1,6 diaminohexane, mix, and rotate as in **step 4** for 30 min. Centrifuge at 3000 × *g* for 2 min, remove all but 10 μL of supernatant, and resuspend the beads.
7. Wash four times with pH 8.4 buffer and resuspend the amino beads into a total of 90 μL of pH 8.4 buffer. We derivatize six sets of beads at a time and leave the six sets overnight at this stage. The amino site density can be measured in a pilot assay to ensure optimal conversion of carboxyl- to amino-terminal groups (*see* Note 3).
8. Add 10 μL of 200 mM sSMCC in DMSO, mix, rotate as in **step 4** for 30 min while the site density of the crosslinker’s maleimide builds on the beads, centrifuge, and resuspend the beads in 10 μL.
9. Wash with 100 μL of pH 7 buffer and resuspend to 360 μL with pH 7 buffer.
10. Prepare and test the nitrogen-bubbling apparatus to give a slow series of bubbles (*see* Note 4).
11. Add 2 μL of 100 mM EDTA and 20 μL of 200 mM glutathione, pH 7, and bubble nitrogen slowly through the suspension for 2 min to remove most of the oxygen. Cap the tube and rotate it slowly for 30 min.
12. Centrifuge at 3000 × *g* for 2 min, remove all but 10 μL of supernatant, and resuspend the glutathione beads. Wash beads four times in the storage buffer of your choice, reducing the concentration of glutathione from 20 mM to below 2 μM. Add 1 mM EDTA and 0.02% sodium azide in the storage buffer to inhibit bacterial growth. Store at 4 °C at a concentration of 10^8^ beads/mL. The beads have been stable for over 2 years. A portion of each bead set is diluted 10× in a storage buffer for ease of assay. Each assay uses 10^4^ beads per target GTPase or 1 μL of diluted beads.
13. It is useful to quantify the number of GST-binding sites on the newly functionalized beads using commercial Quantum™ FITC MESF (*see* Fig. 2) or other methods [29, 30]. Assay the beads by incubating them in 25 nM GST-GFP for 30 min. Use 100 μM soluble glutathione to determine nonspecific binding of GST-GFP to the glutathione beads. Using Quantum™ FITC MESF beads, the glutathione beads synthesized as described support >1.0 million GST-binding sites. At high surface density (>1300 fluorophores/μm^2^), fluorophores on a bead surface undergo self-quenching with increasing site occupancy [31]. It is therefore likely that the 1.0 million sites determined by the calibration beads are a lower limit. However, this measure is useful for tracking the useful shelf life of the beads.

### 3.2 Production of a Cleared Cell Lysate

1. Two days before an assay seed a 48-well plate with 20,000 target cells in 100 μL of culture medium per well, resulting in about 50,000 cells the next day. The rate of cell proliferation might vary based on cell type and conditions. The critical target is 50,000 cells at the start of an experiment.
2. Remove the culture medium and replace with 100 μL of serum-free medium overnight. Specific inhibitors of signaling can be added to the cells for the desired amount of time depending on the requirements of the reader’s assay to establish proper inhibition before stimulation.
3. After stimulating the cells (*see* Subheading 3.5 for examples), chill the plate in an ice/water bath. Add 100 μL of cold 2× RIPA buffer to each well with a 1 mL pipette and triturate the mixture gently to achieve homogenous lysis of the cells.
4. Transfer the lysate into a 0.65 mL microfuge tube and centrifuge at 3–5000 × *g* for 2 min. The 200 μL of cleared lysate is enough for triplicate assays using 50 μL for each test.

### 3.3 Molecular Assembly of GST Effector Proteins on Glutathione Beads

Briefly, glutathione beads are mixed with the desired, purified GST-effector protein of desired concentration (Eq. 1), incubated with rotation overnight at 4 °C, collected by centrifugation, and resuspended in HPSMT buffer to 10,000 beads/μL. Beads are prepared fresh for each experiment and kept on ice until use on the same day. It is desirable to use the beads as a limiting reagent. In this way, uniform site occupancy (*θ* in Eq. 1) of the GSH sites is assured for each bead preparation. The equilibrium dissociation constant (*K*_d_ ~ 80 nM) [26] measured for GST-GFP is used to estimate the occupancy of GSH sites on beads according to Eq. (1):

(1) $$\theta =\left({\left[\text{GST effector}\right]}_{\text{free}}/K_{\text{d}}\right)/\left(\text{1}+\left({\left[\text{GST effector}\right]}_{\text{free}}/K_{\text{d}}\right)\right)$$

Site occupancies of ligand-binding sites at saturation are in the range of 1–4 × 10^6^ ligand sites/bead. Equation (1) can be used to estimate ligand-occupied sites according to the example: 10,000 beads present an upper limit of 4 × 10^10^ sites or 3.3 nM in 20 μL. Incubating 800 nM (10× *K*_d_) GST ligand with 10,000 beads is expected to yield an occupancy, *θ*, of 0.91 (or 91%).

1. 12–18 h before a putative assay of 12 samples for 5 GTPase targets perform the following:
  - Mix 12 μL (12 × 10^4^ beads) of each set of 700 nm color-coded glutathione beads with a tenfold excess (120 μL) of HPSMT blocking buffer #1 for 20 min at room temperature, to block the nonspecific binding sites on the particles.
  - Centrifuge the beads at 3–5000 × *g* for 2 min, our standard.
  - Resuspend each of the five sets in 15 μL of the residual buffer. All further operations are performed at 0–4 °C.
2. Add five distinct GST-effector proteins (800 nM) separately to the five particle sets, where specific effector proteins are associated with the 700 nm intensity register of the bead. Mix the suspensions gently with a nutator overnight. Centrifuge at 3–5000 × *g* for 2 min to reduce the supernatants to 5 μL.
3. Wash the bead sets. For this, add 50 μL of HPSMT to each set and mix, centrifuge at 3000 × *g* for 2 min to reduce the supernatants to 5 μL, resuspend the pellet, and dilute it in 50 μL of HPSMT, giving about 8 nM GST-effector. The beads are ready for use and can be stored at 4 °C overnight.

### 3.4 Assay Runtime

1. Just before the addition of lysates, mix the five different effector beads, centrifuge at 3000 × *g* for 2 min, and reduce the supernatant to about 5 μL. Resuspend the beads in 65 μL of HPSMT, giving approximately 0.8 nM of each GST-effector. Add 5 μL of this suspension to twelve 0.65 mL microcentrifuge tubes for 12 multiplex assays. Leftover beads can be used later to set up the cytometer.
2. Add 50 μL of a cleared lysate to each of the 12 tubes, mix, and rotate for 1 h. When prepared as in Subheading 3.2, there is enough of each lysate for triplicate determinations.
3. Centrifuge the tubes at 5000 × *g* for 2 min, reduce the supernatant to about 5 μL, and resuspend the beads in the residual volume.
4. Wash the beads with 50 μL of blocking buffer #2 to block the beads’ nonspecific antibody-binding sites, centrifuge at 3000 × *g* for 2 min, and resuspend in 5 μL of residual buffer (*see* Note 5).
5. Add 50 μL of primary antibodies (1:20 dilution) against the 5 GTPases to each tube, mix, and rotate the tubes for 1 h.
6. Wash the beads with 50 μL of blocking buffer #2, centrifuge at 3000 × *g* for 2 min, and resuspend in 5 μL of residual buffer. Add 50 μL of goat anti-mouse antibody fluorescently labeled with Alexa488 (1:100 dilution) to each tube, mix, and rotate the tubes for 1 h.
7. Centrifuge the tubes at 3000 × *g* for 2 min, reduce the supernatants to 5 μL, and resuspend the beads in the 5 μL of residual buffer.
8. Dilute the beads from each tube with 100 μL of blocking buffer #1 just before each flow cytometric reading.

### 3.5 Applications of G-Trap Assay

#### 3.5.1 Single-Target Format Measurement of GTP Loading of Rab 7 Associated with the Trafficking of EGF Receptors in EGF-Stimulated Cells

The single-target format of this assay recapitulates published data [16]. The example is shown here as a simplified example of the assay that shows quantification of the active GTPase-occupied sites on the beads using Quantum FITC™ MESF beads. In this experiment, HeLa cells were stimulated with EGF ligand for 10 min and then lysed [16]. Cell lysates of resting and EGF-activated cells were probed with RILP effector beads. One set of beads had no GST-effector protein, and was used to measure nonspecific binding; this is subtracted from the appropriate sample readings. The results are shown in Fig. 2.

#### 3.5.2 Sin Nombre Virus Induces Multiple GTPase Signaling Cascades

**Fig. 3:**
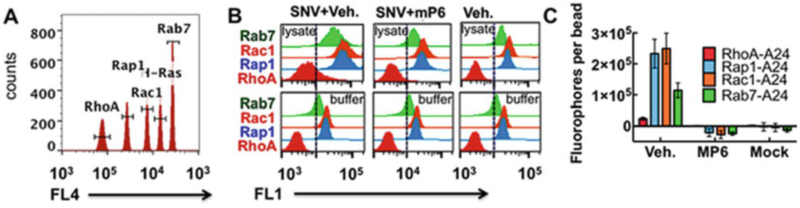
Example output from G-Trap multiplex assay. (**a**) Histograms of a mixture of four Cyto-Plex™ bead populations, identified by their red fluorescence address. Each bead is functionalized with an effector molecule for Rac1 (PAK-1 RBD), H-Ras (RAF RBD), Rho A (Rhotekin-RBD), Rap1 (Ral GDS-RBD), and Rab 7 (RILP-RBD). Gates are used to select beads associated with effector proteins labeled with green fluorescent antibodies shown in panel **b**. (**b**) Top panels show histograms of beads incubated with CHO-A24 cell lysates derived from cells treated with SNV and vehicle (0.1% DMSO), 250 μM DMSO-solubilized mP6 and SNV, and resting cells. Bottom panels represent beads incubated in cell lysis buffer to determine nonspecific binding of anti-GTPase antibodies. (**c**) Respective plots of site occupancy/bead of active GTPases, established from Median Channel Fluorescence (MCF) of histograms shown in b after correction for nonspecific binding. Quantum™ FITC MESF were used to analyze the data shown in b. The error bars represent triplicate measurements for each target. Error bars represent standard deviation. Reproduced from ref. [17] with permission

Integrins are cell adhesion receptors that signal bidirectionally (“inside-out” and “outside-in”) across the plasma membrane [32]. Inside-out signaling stimulates increases in the ligand-binding affinity of integrins [33]. Outside-in signaling by integrins occupied by immobilized ligands [34, 35] induces cell spreading, retraction, migration, proliferation, and survival [32, 35]. Integrin signaling requires both heterotrimeric and monomeric small GTPases [37]. Many viruses engage cellular receptors such as integrins to transit the plasma membrane by hijacking the intrinsic endocytic pathways of desensitizing receptors [38, 39]. Recent studies from our lab have shown that SNV engages the β_3_ integrin plexin-semaphorin-integrin (PSI) domain and initiates integrin outside-in signaling downstream of Gα_13_ activation [17]. In the setting of SNV engagement, outside-in signaling stimulates cytoskeletal remodeling, receptor clustering, internalization, and trafficking [17]. The signaling events involved GTP loading of several GTPases associated with integrin activation (Rap1) [40], cytoskeletal remodeling (RhoA, Rac1) [41], and cargo trafficking (Rab7) [42]. Here we highlight the use of the glutathione bead sets (synthesized as detailed in Subheading 3.1) in a multiplex assay of RhoA, Rap1, Rac1, and Rab7 (*see* Fig. 3a) to determine the signaling outcome of SNV-induced outside-in signaling as previously established [17]. We also use a myristoylated peptide (Myr-$32#-FEEERA-OH) called mP6 [43] to inhibit Gα_13_-dependent outside-in signaling caused by SNV [17]. In this setting there are three different experimental conditions (*see* Fig. 3b). 50,000 CHO-A24 cells in 48-well plates are treated with mP6 or with 0.1% DMSO (solvent for mP6) for 30 min. UV-inactivated particles of Sin Nombre virus (SNV) are added and the cells are incubated for 5 min at 37 °C. The cells are lysed and GTP loading of four GTPases is measured as described in Subheadings 3.2–3.4. Lysis buffer is used to determine the aggregate nonspecific binding of the four reporter antibodies used to detect GTPases associated with the beads. As shown in Fig. 3b, blocking the interaction between the β_3_ integrin cytoplasmic tail and Gα_13_ with mP6 inhibits GTP loading of all GTPases associated with integrin activation (Rap1), cytoskeletal remodeling (Rac1), and trafficking (Rab7). The reader is referred to ref. [17] for the rationale and further details. The top histograms are derived from lysates of cells treated with UV-inactivated SNV, whereas the bottom histograms are derived from beads incubated in RIPA buffer alone, and serve as a measure of nonspecific binding. The quantitative data are shown in Fig. 3c [17].

#### 3.5.3 GTPase Signaling Downstream of Protease-Activated Receptors

Here we use the G-Trap assay to measure in parallel RhoA·GTP, Rac1·GTP, and Rap1·GTP levels in endothelial cells exposed to plasma samples from *de-identified* subjects previously hospitalized for hantavirus cardiopulmonary syndrome (HCPS). Sample use was approved under UNM IRB#15–166. The bead sets were prepared as described in Subheading 3.3. The assay required five sets of beads for the conditions of the experiment and was performed in a single afternoon (≤4 h).

**Fig. 4:**
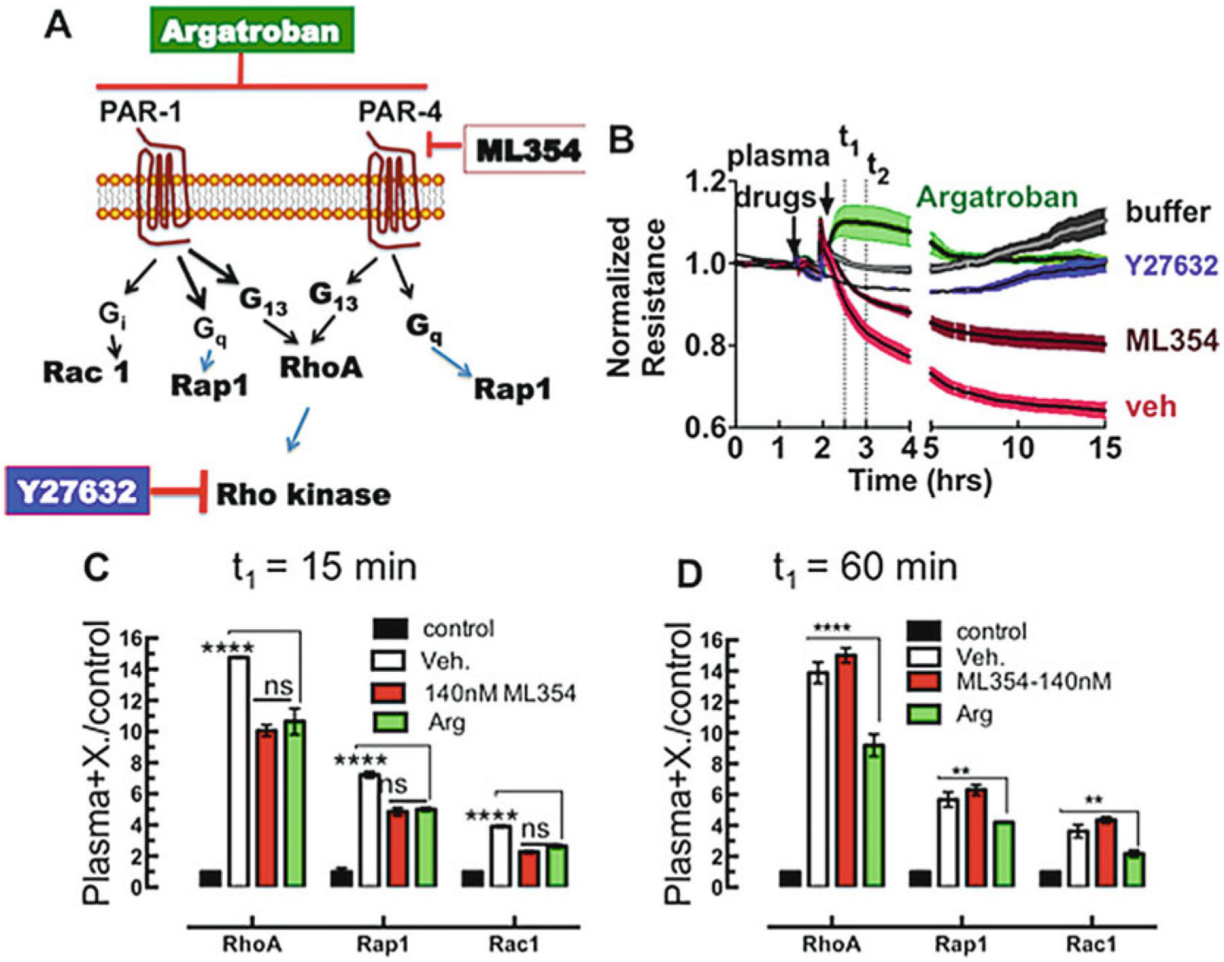
RhoA activation stimulates loss of cell barrier function. (**a**) Model of protease-activated receptor-1 and −4 (PARs) coupling to multiple G proteins (Gα_i/o_, Gα_q_, and Gα_12/13_), upstream of small GTPase activation. Activation of PARs results in cell barrier disruption (RhoA) whereas Rac1 and Rap1 signaling are believed to be barrier protective [57–58]. Argatroban is an orthosteric inhibitor of thrombin interaction with PARs and is barrier protective. ML354 is a specific inhibitor of PAR4 activation. (**b**) Electric cell-substrate impedance sensing (ECIS) measurement of the effects of plasma from a patient with hantavirus cardiopulmonary syndrome (HCPS) on the cell barrier function of telomerase-immortalized microvascular endothelial (TIME) cell monolayers. Inhibitors are added to cell monolayers 30 min before the plasma is added as indicated by arrows and barrier function is measured. (**c**) GTPase activity measured in TIME cells after *t*_1_ = 15-min exposure to HCPS plasma. The results are corrected for nonspecific binding and normalized to resting cells. GTP loading increases in plasma treated cells. Drugs limit the increase in GTP loading compared to untreated cells. GTP loading in cells treated with ML354 is comparable to argatroban treatment. Cell barrier disruption is consistent with a significant increase in GTP loading in RhoA. Each multiplex data point was measured using effector beads prepared as described in Subheading 3.3. (**d**) After 1-h exposure to plasma, RhoA activity is observed in ML354 but not argatroban-treated samples. GTP loading is consistent with cell barrier disruption shown in the ECIS time course. The multiplex beads used for this dataset were similar to panel **c**, for cells lysed after 1-h exposure. Error bars are standard deviation. *P < 0.05; **P < 0.01, ****P < 0.0001 by Dunnett’s *t*-test

Thrombin activates PARs and causes loss of cell barrier function [44–47]. G_12_/G_13_–RhoA·GTP–MLCK (myosin light chain kinase) and Gi-Rac·GTP (Gq-Rap1·GTP) signaling axes are cytoskeletal altering pathways that control cell contraction and spreading, respectively (*see* Fig. 4a). These signals ultimately combine to induce profound changes in vascular endothelial cells, including increased endothelial monolayer permeability [44, 47, 48]. High concentrations of thrombin expressed in the circulation of HCPS subjects significantly contribute to loss of cell barrier function in endothelial cells [18]. Argatroban, an orthosteric inhibitor of thrombin (*K*_i_ ~ 10^−8^ M), can be used to block thrombin activity [49]. Because GTP loading of RhoA is associated with loss of cell barrier function, a set of beads were used to interrogate lysates of cells treated with an inhibitor of Rho kinase (Y27632) before exposing them to plasma samples. In a pilot study of a specific PAR4 antagonist, ML354 [50], we tested the G-Trap platform to determine the role of GTP loading of RhoA, Rap1, and Rac 1 on cell barrier function using telomerase-immortalized microvasculature endothelium (TIME) cells [17]. We first assayed the effects of plasma and PARsignaling inhibitors in terms of the status of cell-cell barrier integrity using electric cell-substrate impedance sensing (ECIS). We then correlate changes in cell barrier function to a time-course measurement of GTPase activity. As shown in Fig. 4b, ECIS measurements show that HCPS patient plasma caused loss of cell barrier function in TIME cells. The cell barrier function of Y27632-treated cells was conserved, consistent with normal activation of RhoA. ML354 treatment conferred only short-term barrier protection and argatroban supported long-term cell barrier integrity to cell monolayers (*see* Fig. 4b). We measured GTP loading of RhoA, Rap1, and Rac1 in a multiplex format. The G-Trap assays show that the short-time (15 min) exposure of cells to plasma increased RhoA·GTP 15-fold, Rap1·GTP 7-fold, and Rac1·GTP 4-fold relative to active GTPase levels in resting cells (*see* Fig. 4c). ML354 and argatroban limited GTP loading to ten-, five-, and threefold for RhoA, Rap 1, and Rac 1, respectively, in the short term. However, after 1-h exposure to plasma, the efficacy of ML354 at limiting GTP loading to the target GTPases is lost, while the activity of argatroban is conserved (*see* Fig. 4d). These assays demonstrate the utility of the G-Trap assay in easily connecting functional (ECIS cell barrier sensing) and mechanism (GTP loading primarily to RhoA).

#### 3.5.4 Testing Septic Patient Plasma for GTPase Activity

Bacteria overcome host defenses, by hijacking Rho GTPases that regulate the actin cytoskeleton [11, 12, 51]. During initiation of infection, bacterial adhesins favor tissue colonization, whereas, at later stages, exotoxins promote bacterial spread and blockage of immune cell responses [52]. By downregulating Rho GTPases, bacterial pathogens can block crucial immune cell functions such as chemotaxis, phagocytosis, and antigen presentation [12, 53]. Bacteria produce various toxins and virulence factors that activate or inactivate Rho GTPases by different mechanisms. The processes include (a) posttranslational modification of the GTPases; (b) bacterial protein mimics of GTPase regulatory factors, including guanine nucleotide exchange factors (GEFs), GTPase-activating proteins (GAPs), and guanine nucleotide dissociation inhibitors (GDIs); and (c) modification of upstream regulators of Rho GTPases [1, 12].

**Fig. 5:**
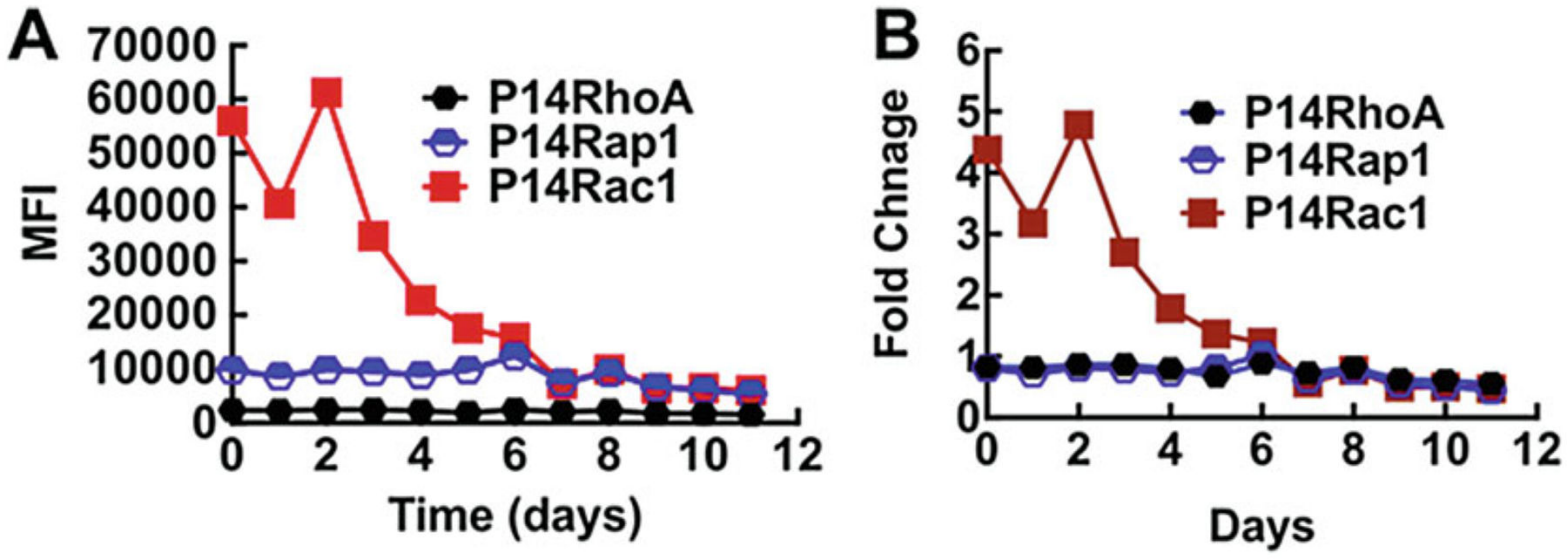
GTP loading of RhoA, Rap1, and Rac1 measured in TIME cell lysates after 30-min exposure to serial plasma samples drawn from a septic patient (P14) 0–12 days after hospital admission for sepsis. The patient underwent gastric surgery after day 5 when the bacterial infection was brought under control. The sterile inflammation resulting postsurgery did not elicit any further spike in GTPase activity. (**a**) Raw G-Trap data for a patient who tested positive for bacterial infection (*S. pneumoniae*) on day 0, and was treated with antibiotics. (**b**) Data normalized to GTP loading of each GTPase as measured on day 11

Here we test the applicability of the G-Trap assay for detecting bacterially induced GTPase activity in serial plasma samples from a de-identified septic patient (UNM IRB #13–312). The patient was diagnosed with community-acquired pneumonia (*S. pneumoniae* on hospital admission) and treated with antibiotics. Vero E6 cells from a cell culture were treated with 10 μL of plasma samples/test. Following a 30-min incubation, Vero cell lysates were prepared as described in Subheading 3.2 and GTP loading of Rho A, Rac1, and Rap1 were simultaneously measured in each lysate using a mixture of PAK-1 RBD, Rhotekin-RBD, and Ral GDS-RBD beads. As shown in Fig. 5, the plasma samples added to Vero E6 cells in culture elicit GTP loading principally of Rac1 during the first 4 days after hospital admission. After that, plasma levels of the Rac1-activating factor decreased in response to antibiotic treatment and Rac1 GTP levels reverted to basal levels similar to RhoA and Rap1, which were unchanged across the entire time course of patient hospitalization. The selective activation of Rac1 by blood plasma collected during the infectious phase is consistent with the fact that *S. pneumoniae* produces a toxin, pneumolysin, which activates Rac1 GTPases [54, 55]. Serial samples from patients with sterile inflammations indicated basal GTPase activity only (not shown). It is also interesting to note that on day 5 the patient underwent stomach surgery, which did not elicit overt GTPase activity. These results illustrate the potential utility of the G-Trap assay for diagnostic purposes using serial samples.

## 4 Notes

1. The site density of glutathione sites on beads governs the magnitude of the fluorescence signal from GST proteins bound to the beads. A low site density of GST sites on beads can yield variable data or poor binding results [28]. Derivatization of the carboxyl Cyto-Plex™ beads to glutathione requires an intermediate step of functionalizing to amino groups. Optimizing the synthesis of amino groups is essential. Derivatizing the amino-terminated groups with a fluorescent probe such as NHS-Alexa 488 tests optimum conversion of carboxyl to amino groups. For this purpose, it is useful to use inexpensive carboxyl-functionalized beads such as those from Spherotech (*see* Note 3). Glutathione derivatization is tested with 25 nM GST-GFP for 30 min as described in Sub-heading 3.1, **step 13**.
2. All buffers contain 0.01% Tween-20, which is compatible with most biological molecules.
3. To test the conversion efficiency of carboxyl beads to amino beads, we derivatized 10 μL of generic carboxyl beads (Spherotech) to amino beads. We use commercial amino beads of similar size with known amino group site density for comparison, with our synthesis. The two amino bead sets are then reacted with NHS-Alexa488 in parallel. Approximately 0.1 mg of NHS-Alexa488 is dissolved in 20 μL of dry DMSO to give about 5 mg/mL, which is stored at −80° C. Ten thousand synthesized amino beads and ten thousand commercial amino beads are put in 20 μL of pH 8.4 buffer, 2 μL of NHS-Alexa488 solution is added, the suspension is mixed, and reagents are allowed to react for 30 min in the dark. The beads are washed twice with pH 7 buffer, diluted to 50 μL of buffer, and analyzed by flow cytometry. We determine nonspecific binding of NHS-Alexa488 to beads by mixing carboxyl beads with the fluorophore. In our setting the fluorescence from the nonspecific attachment of NHS-Alexa488 to carboxyl beads was 20% of the conjugated fluorophores. Our amino beads were comparable to the commercial beads.
4. Bubbling nitrogen slowly through 400 μL of suspension in a 1.6 mL microfuge tube is not easy. We use a narrow nitrogen line and very low nitrogen pressure, and notice that the angle of the tube of suspended beads matters: tipping the slowly bubbling microfuge tube from horizontal to upright can stop bubbling, probably due to increased hydrostatic pressure. Another technique to prevent bubbling is to use a soft nitrogen tubing line, which can be pushed against the bottom of the centrifuge tube to stop bubbling. Tween-20 gives an observable bubble running up the microfuge tube, and we estimate that the volume of air above the suspension is displaced about ten times during the 2 min of bubbling.
5. Blocking of nonspecific binding sites for primary and secondary antibodies with BSA is critical for limiting nonspecific binding. It is also important to test new antibodies in single-target format before using in a multiplex format. In our experience new antibody batches from “trusted sources” can be highly nonspecific, and could bind to all bead surfaces regardless of effector functionalization, and either raise the background intensity for all beads in the multiplex assay or at worst degrade the readout of all the beads in a multiplex configuration.

## Figures and Tables

**Table 1:** Reagents, concentrations, and conditions used for illustrative experiments [16]

GTPase	Effector	Activator; final concentration; incubation time	Inhibitor; final concentration; incubation time	SNV particle titer; incubation time
RhoA	Rhotekin RBD	Calpeptin; 1 μM; 30 min		10,000/cell; 3, 10, 20, 30, 60 min
Rac1	PAK-1 PBD	EGF; 10 nM; 15 min	NSC23766; 100 μM; 30 min	10,000/cell; 3, 10, 20, 30, 60 min
Rap1	Ral-GDS RBD	8-Cpt-2me-cAMP; 50 μM; 30 min	GGTI 298; 10 μM; 30 min	10,000/cell; 3, 10, 20, 30, 60 min
R-Ras	Raf-1 RBD		FTI-277; 100 nM; 30 min	
H-Ras	Raf-1 RBD		FTI-277; 100 nM; 30 min	
Rab7	RILP RBD	EGF; 10 nM; 15 min		10,000/cell; 3, 10, 20, 30, 60 min
